# Role of exosomes in pathogenesis, diagnosis, and treatment of diabetic nephropathy

**DOI:** 10.1186/s12882-025-04120-4

**Published:** 2025-05-08

**Authors:** Shaimaa I. Barr, Eman M. Abd El-Azeem, Sahar S. Bessa, Tarek M. Mohamed

**Affiliations:** 1 Biochemistry Department, Faculty of Science, Ain Shams University, Cairo, Egypt; 2 Internal Medicine Department, Faculty of Medicine, Tanta University, Tanta, Egypt; 3 Biochemistry Division, Chemistry Department, Faculty of Science, Tanta University, Tanta, Egypt

**Keywords:** Diabetic nephropathy, Exosomes, Biomarker, Pathogenesis, Therapeutic strategies

## Abstract

Diabetic nephropathy (DN) is a serious microvascular complication that can progress to end-stage renal disease, with its prevalence and associated mortality increasing globally. However extensive research, the precise mechanisms underlying DN pathogenesis remain unclear, and the current treatment options for DN are limited to dialysis or renal replacement therapy, although several experimental approaches have shown potential, they remain investigational and lack clinical translation. Exosomes play a pivotal role in disease diagnosis and prognosis. Urinary exosomes, originating from various kidney cells, reflect the kidney’s pathological condition and are involved in cell-to-cell communication through autocrine or paracrine signaling; therefore, they could contribute to the pathogenesis of DN and potential therapeutic approaches. Additionally, due to their diverse cargo, which depend on cellular origin and pathological state, exosomes may act as biomarkers for the early prediction of DN. This review presents a comprehensive overview of the latest findings on the role of exosomes in the diagnosis, pathogenesis, and treatment of DN.

## Background

Diabetic nephropathy (DN) represents the foremost microvascular complication, which induces damage to both glomerular and tubular cells, leading to end-stage renal disease (ESRD). DN is pathologically characterized by glomerular hypertrophy, sclerosis, interstitial fibrosis, and hyperfiltration [1, 2]. Despite ongoing research, the mechanism of DN pathogenesis is not fully elucidated; however, the pathogenesis of DN involves collaboration between hemodynamic and metabolic factors [3, 4]. The metabolic factors cause production of advanced glycation end products (AGEs) as hyperglycemia induce glycation of protein, lipid, and nucleic acid, leading to increased release of profibrotic cytokines and increasing reactive oxygen species (ROS) in renal cells [4, 5]. Additionally, the hemodynamic factors cause intraglomerular hypertension resulting in glomerular injury. The hemodynamic factor is caused by up-regulation of renin-angiotensin system (RAS) which is mediated by hyperglycemia [4]. These two pathways act to elevate expression of chemoattractant and adhesion molecules begins to attract inflammatory cells to kidney tissues, promoting hyperfiltration, sclerosis, and acting as a mediator for further inflammatory responses [3]. Approximately one-third of type 1 and about 50% of type 2 diabetic patients will develop DN. Among these, about 50% of DN cases will ultimately progress to ESRD that can’t be reversed and requires renal replacement therapy, the only available treatment [3]. Several biomarkers have been proposed for the diagnosis of DN, such as glomerular filtration rate (GFR) and albumin-to-creatinine ratio (ACR). However, these biomarkers lack sensitivity and specificity as it has been observed advanced damage in kidney filtration slits without changes in the urinary albumin level. Therefore, the identification of early diagnostic biomarkers and the pathogenesis mechanism of DN is mandatory for better disease management and to recognize effective treatment strategies to reduce the progression towards ESRD [6, 7].

Extracellular vesicles (EVs) are lipid bilayer structures that detach from cells and are divided into two major groups based on their biogenesis and size range: microvesicles (MVs) with a diameter (150–1000 nm) and exosomes with a diameter (30–150 nm)originating from the endosomal pathway via multivesicular bodies. Moreover, they can be classified according to size, density, sedimentation force and specific biomarkers that exist in the vesicle [8]. However, it is important to note that the term ‘exosomes’ is often used interchangeably with other small EVs in the literature as according to MISEV 2023 (PMID: 38326288), achieving pure isolation of exosomes from biological fluids remains highly challenging due to the overlap in size, density, and composition with other extracellular vesicles (EVs), such as microvesicles and smaller particles. Most used isolation techniques, including ultracentrifugation, size-exclusion chromatography, and precipitation-based methods, often result in heterogeneous EVs populations rather than highly purified exosomes [9].

Exosomes are a subset of EVs, they are promising biomarkers for disease diagnosis due to their rich cargo, which includes protein, metabolites, and nucleic acid (mRNA, DNA, and miRNA). Furthermore, exosomes are present in several body fluids, such as blood, urine, saliva, breastmilk, cerebrospinal fluid, amniotic fluid, and ascitic fluid [10].

Exosomes participate in cell-to-cell communication by transferring genetic material from one cell to another, as they can be transported through the circulatory system to distal sites, not just the immediate cell area [11, 12]. Moreover, exosomes can serve as diagnostic biomarkers for various diseases, including tumors such as prostate, breast, and ovarian cancer, as well as glioblastoma [13–16]. Additionally, they enable the early detection of Alzheimer’s disease by early detection of beta amyloid peptide associated with exosomes [17]. Furthermore, exosomes isolated from urine have been associated with several kidney pathologies, including renal ischemia [18]. Exosomes also contribute to the development of novel therapeutic strategies by enabling RNA modification and delivering it to target cells [19]. However, exosomes also play a role in developing several diseases, such as cardiovascular disease, neurodegenerative diseases, and cancer metastasis, by transferring signals and reprogramming target cells. Furthermore, they have a role in DN pathogenesis by inducing renal injury, apoptosis, inflammation, and fibrosis [20, 21].

The main objective is to investigate the cargo of trans-sorted active molecules, such as mRNAs, proteins, and other bioactive components, within the vesicles. This involves understanding their functional roles in cellular communication, signaling, or pathological processes. The content of vesicles varies according to the cells’ origin and the pathological stage. Therefore, the identification and analysis of the specific active molecules of vesicles reflect a complete overview of the state of the origin of vesicles and may hold diagnostic or therapeutic significance.

## Biogenesis of EVs

### ***Microvesicles (MVs)***

**Fig. 1:**
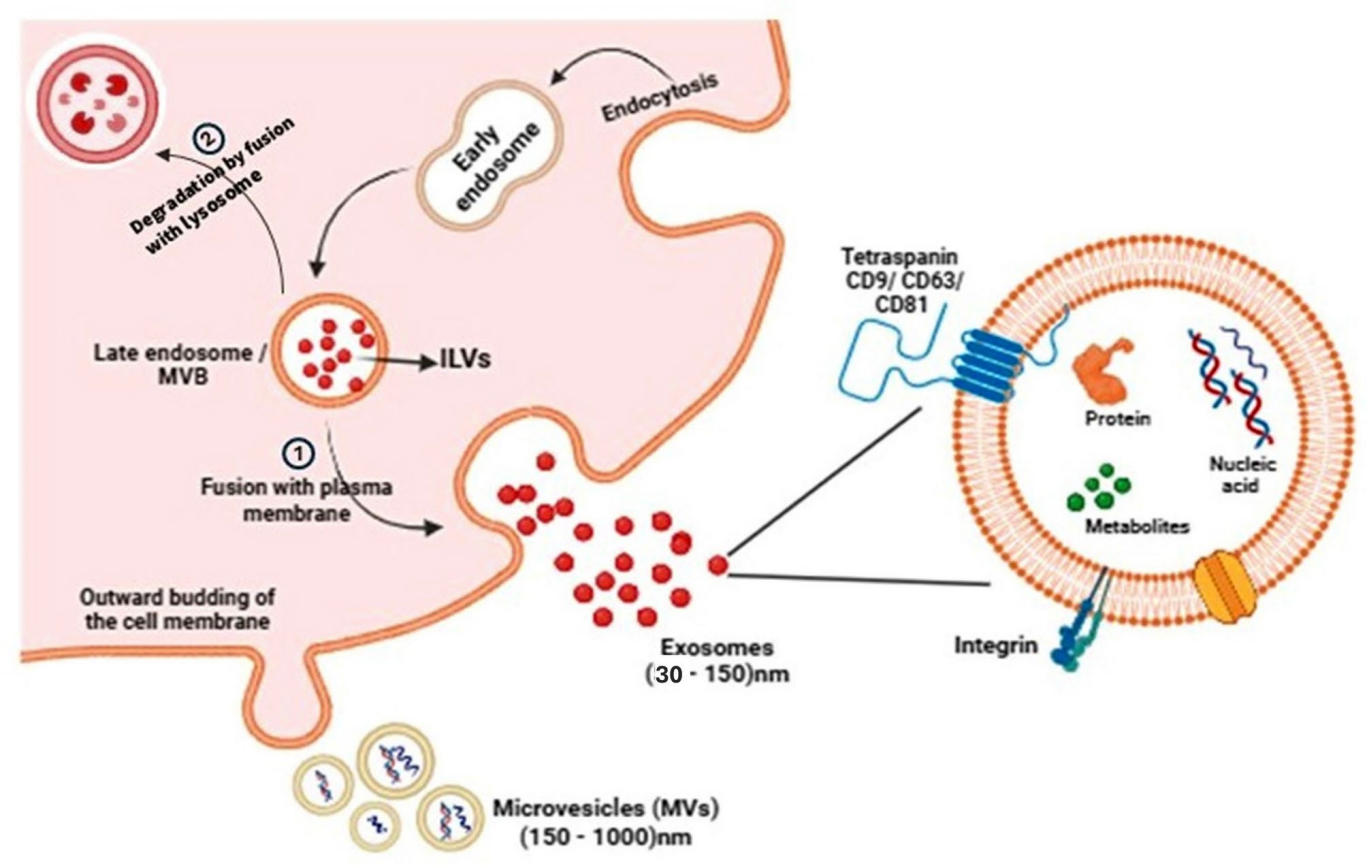
Biogenesis of extracellular vesicles (exosomes, microvesicles). *Exosomes*, (30–150) nm in diameter, are produced by the budding of early endosomes into the lumen, forming MVBs, which contain several ILVs. MVBs have two fates: 1-fusion of MVBs with the plasma membrane, and the ILVs are released into the extracellular space as exosomes, or 2-degradation by fusion of MVBs with lysosomes. *Microvesicles (MVs),* (150–1000) nm in diameter, are produced by direct outward budding of the cell membrane. Created in https://biorender.com

MVs are formed by shedding the outward budding of the plasma membrane; they are formed by the interaction between redistributed phospholipid and contraction of cytoskeletal protein. This process is initiated by amino-phospholipid translocases (floppases) that translocate the phosphatidylserine from the inner leaflet to the outer leaflet membrane and accomplished by the contraction of the actin-myosin system [22]. Contraction of membrane commences by the activation of ADP-ribosylation F6 (ARF6) that leads to the activation of phospholipase-D (PLD), followed by the attraction of extracellular signal-regulated kinase (ERK) to the plasma membrane, which activates myosin light chain kinase (MLCK) by phosphorylation, which activates myosin light chain and results in the formation of MVs [22] (Fig. 1).

### ***Exosomes***

Exosomes are formed by inward budding of the plasma membrane, forming early endosomes, then forming the multivesicular bodies (MVBs) or late endosomes that contain several intraluminal vesicles (ILVs) [10]. The fate of these late endosomes can be either fusion with lysosomes, leading to the destruction of their contents, or fusion with the plasma membrane, resulting in the release of exosomes (ILVs) into the extracellular space, regulated by Rab GTPases which facilitate the fusion of the MVBs with the plasma membrane [22]. ILVs are formed by the inward budding of the MVB membrane.This process is complex and can occur through multiple pathways, including ESCRT-dependent and ESCRT-independent routes [10]. The biogenesis includes proteins of the endosomal sorting complex required for transport (ESCRT), which is essential for ILV formation and is involved in sorting ubiquitinated molecules for engulfment and divided into four groups (ESCRT-0, I, II, III), an accessory protein called ALG-2 interacting protein (ALIX), and tumor susceptibility gene (TSG 101) [19, 22].

ESCRT-0 has an important role in MVBs formation as it consists of signal transduction adaptor molecule-1 protein (STAM 1) that identify ubiquitinated protein on the endosomal membrane and forms clusters then these proteins are taken into endosome forming MVBs. ESCRT-I, II are required for deformation membrane into buds, ESCRT-III is responsible for the separation of the bud, and it is recruited by ALIX this protein bind to TSG 101, which is a component of ESCRT-I [22, 23] (Fig. 1). Also, the MVBs/ILVs can be formed by ESCRT-independent mechanisms include lipid as ceramide or protein as tetraspanin proteins can influence exosome biogenesis and cargo sorting [11, 24]. Ceramide produced by lipid metabolism enzymes neutral sphingomyelinase (nSMase) that hydrolyses sphingomyelin into ceramide which participate in inward budding and the formation of ILVs [25]. Phospholipase D2 (PLD2) hydrolyses phosphatidylcholine into phosphatidic acid that controls the budding of MVBs and the exosomes development by acting as effector protein of ARF6 [26]. Tetraspanins organize membrane microdomains by forming clusters and interacting with a wide range of transmembrane and cytosolic signaling molecules. These domains, known as tetraspanin-enriched microdomains (TEMs), serve as platforms for cargo transport [27, 28]. Tetraspanins, CD9, CD63, and CD81 are highly present in exosomes, are often used as exosome biomarkers and can influence exosome biogenesis and cargo sorting [29]. CD63 (the lysosomal-associated membrane protein 3– LAMP-3) interact with the melanocyte-specific glycoprotein (PMEL) enters ILVs [30]. Also, it has a crucial role in the incorporation of latent membrane protein 1 (LMP1) in exosomes and enhance vesicle production by enables escape from lysosomal degradation [31]. Moreover, CD9 interacts with metalloproteinase CD10, to enhance exosomal loading of CD10 [32], and CD9 and CD82 interact with E-cadherin to promote the exosome secretion of β-catenin which is a key protein in cell–cell adhesion [33]. Furthermore, exosomes can be formed by syndecan 1-syntenin 1-ALIX pathway [34]. Additionally, CD81 and CD82 regulate the formation an of cell membrane protrusions [35].

### Role of urinary exosomes in pathogenesis of DN

**Fig. 2:**
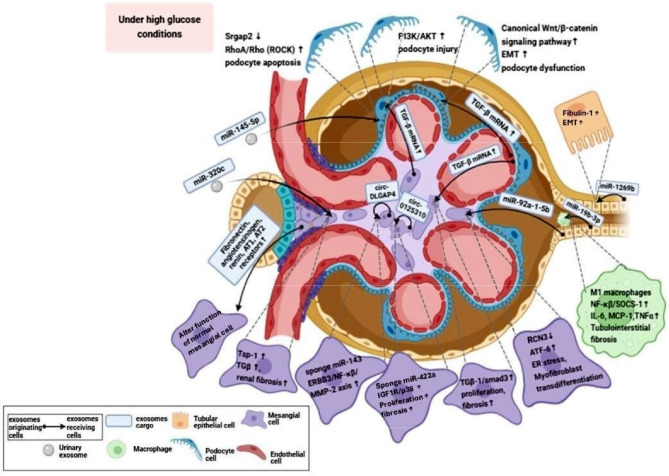
Role of exosomes in pathogenesis of DN. Exosomes transferred from exosome-originating cells to exosome-receiving cells in an autocrine or paracrine pattern, modulate several signaling pathways in the receiving cells due to their cargo. Created in https://BioRender.com

Urinary exosomes are produced from different types of cells within the kidney such as glomerular endothelial cells (GECs), macrophages, glomerular podocytes, mesangial cells (MCs), and tubular epithelial cells (TECs). These vesicles contain protein and genetic material (miRNA, DNA, mRNA), carbohydrates, and lipids. These contents vary according to cells’ origin and pathological state [36]. Exosomes translocated to other cell type in an autocrine or paracrine pattern and influence the pathological change due to their cargo that modulate signaling pathway of the receiving cells [20, 37]. The pathological role of exosomes is illustrated in Fig. 2.

In terms of paracrine communication, research has illustrated that high glucose condition stimulates proximal tubular epithelia cells (PTECs) to produce exosomal miR-92a-1–5p, which is transferred to glomerular mesangial cells (GMCs), where it induces myofibroblast trans-differentiation. Also, exosomal miR-92a-1–5p can dysregulate genes responsible for maintaining endoplasmic reticulum (ER) homeostasis by downregulating reticulocalbin‑3 (RCN3) expression and upregulating activating transcription factor 6 (ATF-6) expression. This dysregulation leads to ER stress and contributes to the progression of DN. This effect can be counteracted by antagomir-92a-1–5p that blocks endogenous miR-92a-1–5p, this treatment induces RCN3 expression, attenuates ATF-6 expression, and reduces mesangial matrix accumulation in the glomerulus [38].

Additionally, tubular epithelial cell (TEC)-derived exosomal miR-19b-3p is internalized by macrophages, leading to activation of macrophages and formation of the M1 macrophage phenotype by the activation of the NF-κB/SOCS-1 signaling pathway, which promotes inflammation and tubulointerstitial fibrosis. This effect can be mitigated by inhibiting miR-19b-3p, which ameliorates NF-κB activation and reverses the upregulation of inflammatory cytokines, including IL-6, MCP-1, and TNF-α [39].

High glucose (HG)-treated GECs secrete more exosomes highly enriched with transforming growth factor beta (TGβ-1) mRNA than normal glucose-treated cells that can reach podocyte cells, leading to epithelial-mesenchymal transition (EMT) and podocytes dysfunction [40]. Additionally, the activation of the canonical Wnt/β-catenin signaling pathway was observed, which promotes proliferation and contributes to the EMT of podocytes induced by exosomes. In the cited study, GECs were cultured under normal glucose (NG; 5.6 mmol/L glucose + 24.5 mmol/L mannitol) or HG (30 mmol/L glucose) conditions for 24 h. The increased expression of exosomal contents, including TGF-β1, Wnt, and β-catenin, was determined by RT-PCR [40].

Moreover, exosomes from HG-treated GECs promote the proliferation and fibrosis of glomerular mesangial cells (GMCs) through the activation of the TGF-β1/Smad3 signaling pathway. This was demonstrated when GMCs were incubated with exosomes derived from GECs cultured under NG (5.5 mmol/L glucose + 24.5 mmol/L mannitol) or HG (30 mmol/L glucose) conditions for 24 h [41]. Fibrosis can be mitigated by inhibiting TGF-β1 released from GEC-derived exosomes, as shown when podocytes were incubated with HG-treated GEC-derived exosomes silenced for TGF-β1 mRNA (HG + siRNA-GEC-Exo) [40]. Additionally, Tongxinluo has been found to inhibit renal fibrosis by suppressing the intercellular transfer of TGF-β1-containing exosomes from GECs to GMCs [42].

Exosomes derived from HG-treated glomerular mesangial cells (GMCs) trigger podocyte injury by activating the TGFβ1-PI3K/AKT signaling pathway. This effect can be treated by berberine that reduces TGβ-1 in HG-treated GMCs-derived exosomes, therefore reducing apoptosis and attenuating podocyte damage which indicated when podocyte cells were co-cultured with exosomes derived from GMCs cultured for 24 h under normal glucose (NG; 5.6 mmol/l glucose + 24.5 mmol/l mannitol), HG (30 mmol/l glucose), or HG plus berberine. The TGFβ1 content in exosomes was quantified using ELISA, while activation of the PI3K/AKT pathway in podocytes was assessed by Western blot analysis [43].

Regarding the autocrine communication, Bai and colleagues illustrated that exosomes isolated from HG-treated MCs or serum of patients with DKD have significantly increased circ_DLGAP4 that promote proliferation and fibrosis of renal MCs by sponge miR-143 and modulating ERBB3/NF-κB/MMP-2 axis leading to DN progression. The cited study demonstrated increased expression of circ_DLGAP4 in exosomes isolated from the serum of DKD patients and cultured MCs under high glucose conditions (25 mM glucose) compared to normal subjects and NG-treated MCs (5.5 mM glucose). Furthermore, the established DKD rat models with circ_DLGAP4 showed that miR-143 level was repressed by circ_DLGAP4, whereas ERBB3 and MMP-2 mRNA levels were increased by circ_DLGAP4. Conversely, exosomal circ_DLGAP4 was downregulated by miR-143 mimic [44]. Additionally, exosomes isolated from high glucose-induced MCs promote MCs proliferation and fibrosis by overexpressing circ_0125310 which sponge the miR-422a and targeting the IGF1R/p38 axis. This effect can be reversed by the knockdown of circ_0125310. Flow cytometry analysis of MCs cultured under normal glucose (5.5 mM) and high glucose (30 mM) conditions confirmed this finding. In vivo studies further demonstrated that the injection of circ_0125310 into a diabetic rat model accelerated DN progression [45]. Furthermore, exosomes isolated from high glucose-treated MCs exhibit elevated levels of fibronectin, angiotensinogen, renin, and AT1 and AT2 receptors, which alter the function of normal MCs [46].

High glucose stimulated TEC-derived exosomes showed high level of fibulin-1 that trigger EMT in TEC. The expression of FBLN1 was modulated by miR-1269b, as the phenotype of TECs toward mesenchymal type. Exosomes derived from HK-2 cells treated with NG (5.5 mM) and HG (25 mM) for 48 h were isolated and incubated with HK-2 cells in normal conditions. The protein content of exosomes was examined by LC-MS and the morphology of HK-2 cells incubated with exosomes changed from the cuboidal epithelial structure to the elongated mesenchymal shape [47].

Other exosomes produced under high glucose conditions, high glucose stimulate the overexpression of miR-145–5p in urinary exosomes, leading to podocyte apoptosis and subsequent loss. These urinary exosomal miR-145–5p are taken up and internalized by mesangial podocyte cells (MPCs), resulting in the inhibition of Srgap2 and activation of the RhoA/Rho kinase (ROCK) pathway. The ROCK pathway plays a crucial role in maintaining the function and structure of various kidney cells and is involved in the regulation of podocyte apoptosis, which is essential for maintaining the integrity of the glomerular filtration barrier. Srgap2 protects podocytes by inactivating RhoA/Cdc42, which inhibits high glucose-induced cell migration. Therefore, dysregulation of this pathway contributes to podocyte injury and loss in DKD. When (45 μg/ml) DKD-Exo that were isolated from the urine samples of patients with DKD (DKD-Exo) were co-cultured with MPCs for 24 h in the presence or absence of microRNA-145–5p inhibitor showed by RT-PCR that miR-145–5p expression was markedly upregulated, negatively regulated Srgap2 levels, and activated ROCK, which was reversed by the presence of the miR-145–5p inhibitor [48]. Additionally, in type 2 diabetes, urinary exosomes showed significant up-regulation of miR-320c that increase TGF-β signaling by targeting thrombospondin-1 (TSP-1), which causes renal fibrosis and correlates with microalbuminuria [49, 50]. As Ingenuity Pathway Analysis (IPA) indicated that miR-320c targets the TSP-1 protein, which is increased in the glomeruli of patients with both type 1 and type 2 DN and correlates with TGF-β activity [49]. Moreover, immunohistochemical staining of kidney tissue samples from DN patients was performed to assess the correlation between glomerular and cortical expression of TSP-1, p-Smad2/3, glomerular fibrosis, and sclerosis [50].

This highlights the diverse origins and cargo of urinary exosomes, emphasizing their role in cell-to-cell communication and their potential contribution to the pathogenesis of DN. Future studies should explore the specific mechanisms by which exosomal cargo influences renal cells and how these processes can be targeted for therapeutic interventions.

### Role of exosomes in therapeutic strategies of DN

There is a growing interest in exosomes as a potential therapeutic agent for DN. The role of exosomes from different sources has been illustrated to ameliorate the pathological state of DN by regulating the pathways involved in renal injury. The therapeutic effect of exosomes in DN is summarized in Table 1.

**Table 1:** Role of exosomes in treatment of DN

Source of exosomes	Model	Cargo	Action	Ref
M2 macrophages	Mouse podocyte cell line	miR-25-3p	Inhibit expression of DUSP, activating podocyte autophagy and attenuating podocyte apoptosis	[51]
PTECs	STZ-induced DN mice	miR-26a-5p	Bind CHAC1, inhibiting the CHAC1/NF-κB pathway, thus inhibits the inflammatory response. The expression of miR-26a-5p in exosomes is increased by inhibiting Rab27a.	[52]
ADSCs	Mouse podocyte cell line	miRNA-215-5p	Attenuate epithelial–mesenchymal transition (EMT) by inhibiting the transcription of ZEB2, thus reducing podocyte loss	[53]
ADSCs	db/db mice	miR-486	Enhance autophagy and reduce apoptosis of MPCs by inhibiting the Smad1/mTOR signaling pathway in podocytes	[54]
ADMSCs	Rat glomerular mesangial cell line, STZ-induced DN Sprague–Dawley rat	miR-125a	Inhibit apoptosis and protect against DN by modulating histone deacetylase-1 (HDAC1) and downregulating endothelin-1 (ET-1)	[55]
MSCs	db/db mice	miR- 424-5p	Inhibit YAP1 activation, thus reducing high glucose- induced apoptosis and reduced EMT. It attenuates DKD progression	[56]
MSCs	Human embryonic kidney epithelial cells (HKCs) injury induced by high glucose (HG)	miR-125b	Induce autophagy and inhibit apoptosis by downregulating TRAF6/Akt signaling pathway	[57]
MSCs	STZ-induced DN albino rats	………..	Induce autophagy and suppression the mTOR pathway, leading to improved renal function and histological restoration of renal tissues	[58]
HUC-MSCs	Podocyte cell line and mice	miR-22-3p	Ameliorate kidney injury by reducing inflammation in podocytes and inhibiting the activation of NLRP3 signaling pathway in podocytes	[59]
HUC-MSCs	STZ-induced DN rats	miR-146a-5p	Inhibit TRAF6/STAT1signaling pathway, thus mediate macrophage shifting polarization from M1 to M2. Reduce inflammatory cytokine level	[60]
HUC-MSCs	STZ-induced DN rats, renal tubular epithelial cell lines (NRK-52E, HK2), and human renal glomerular endothelial cell line (hrGECs).	………..	Reducing pro-inflammatory cytokines (IL-6, IL-1β, and TNF-α) and pro-fibrotic factor (TGF-β), thus inhibit renal interstitial inflammation, fibrosis, improve renal function, and prevent progression of DN	[61]
BMMSC	STZ-induced DN Sprague–Dawley rat	………..	Inhibit JAK2/STAT3 expression, therefore attenuate renal tissue damage in DN patients and improve renal function	[62]
urinary exosomes	STZ-induced DN rats	miR-30, miR-let-7 family, miR-24-3p, miR-23a-3p	Compensate for the loss of protective miRNAs, so provide a reno-protective effect	[63]
USCs-Exo	STZ-induced DN Sprague–Dawley rat model Human podocytes cell line	GFs, angiogenin, BMP-7	Reduce urinary microalbumin excretion, inhibit apoptosis, suppress the caspase-3 overexpression. In vitro, reduce podocyte apoptosis	[64]
USCs-Exo	Human podocytes cell line STZ-induced DN Sprague–Dawley rat model	miR-16-5p	improve podocyte injury by compensate the inhibited miR-16–5p due to high glucose and silencing VEGFA	[65]

M2 macrophages, which play an anti-inflammatory role, produce exosomal miR-25–3p that is delivered to podocytes, thereby reducing podocyte injury. Exosomal miR-25–3p specifically binds to dual specificity phosphatase 1 (DUSP1) and inhibits its expression, promoting podocyte autophagy and attenuating high glucose-induced podocyte apoptosis [51].

Li et al. illustrated that exosomal miR-26a-5p derived from proximal tubular epithelial cells (PTECs) suppressed the inflammatory response. Inhibition of exosomal secretion in PTECs by inhibiting Rab27a increased miR-26a-5p expression in exosomes derived from PTECs. Exosomal miR-26a-5p inhibits the inflammatory response by binding CHAC1, inhibiting the CHAC1/NF-κB pathway, which prevents the inflammatory response in PTECs and delays the progression of DKD [52].

Adipose stem cells (ADSCs)-derived exosomal miRNA-215-5p is delivered to podocytes and attenuates epithelial-mesenchymal transition (EMT) of podocytes by inhibiting the transcription of zinc finger E-box-binding homebox-2 (ZEB2), thus reducing podocyte loss and slowing the development of DKD as conducted by Jin and colleagues [53]. Another study on a rat model showed that ADSCs-derived exosomal miR-486 enhances autophagy and reduces apoptosis of mesangial podocyte cells (MPCs) by inhibiting the Smad1/mTOR signaling pathway in podocytes [54]. Moreover, adipose mesenchymal stem cell (ADMSCs)-derived exosomal miR-125a inhibit apoptosis and protect against DN by modulating histone deacetylase-1 (HDAC1) and downregulating endothelin-1 (ET-1) [55].

Mesenchymal stem cells (MSCs)-derived exosomal miR-424-5p can reverse high glucose-induced apoptosis and reduce EMT by inhibiting Yes-associated protein 1 (YAP1), which induces cell proliferation. Therefore, it could attenuate DKD progression and produce a protective effect in DKD against apoptosis [56]. Additionally, MSC-derived exosomal miR-125b inhibits the progression of DN by inducing autophagy and inhibiting apoptosis through the downregulation of tumor necrosis factor receptor-associated factor-6 (TRAF6)/Akt signaling pathway [57]. Another study by Ebrahim et al. demonstrated that MSC-derived exosomes exert a nephroprotective effect in DN by upregulating autophagy and suppression of the mTOR pathway, leading to improved renal function and histological restoration of renal tissues [58].

A recent study by Wang et al. illustrated that human umbilical cord mesenchymal stem cells (HUC-MSCs)-derived exosomal miR-22-3p ameliorates kidney injury by reducing inflammation in podocytes and inhibiting the activation of the signaling pathway in podocytes. Therefore, the exosomal miR-22-3p provides protection to the podocyte against the inflammation [59]. Another study using a rat model illustrated that HUC-MSCs-derived exosomal miR-146a-5p markedly improves renal function and downregulates renal injury. This improvement is achieved by reducing the local and systemic inflammatory cytokine levels, mitigating the infiltration of the inflammatory cells into kidney tissue, and shifting macrophage polarization from the M1 pro-inflammatory phenotype to the M2 anti-inflammatory phenotype. The shifting polarization process of macrophages is mediated by HUC-MSCs-derived exosomal miR-146a-5p, which inhibits the tumor necrosis factor receptor-associated factor-6 (TRAF6)/signal transducer and activator of transcription (STAT1) signaling pathway [60]. Furthermore, HUC-MSCs-derived exosomes improve the renal function, inhibit renal interstitial fibrosis and inflammation, by reducing pro-inflammatory cytokines (IL-6, IL-1β, and TNF-α) and pro-fibrotic factor (TGF-β) [61].

Bone marrow mesenchymal stem cells derived exosomes (BMMSC-Exos) attenuate renal tissue damage in DN patients, improve renal function, and lower the blood glucose level by inhibiting JAK2/STAT3 pathway activity [62].

A more recent study using a rat model indicated that injection of DN rats with urinary exosomes produces a reno-protective effect. These urinary exosomes compensate for the loss of protective miRNAs, such as miR-30, which ameliorates diabetic nephropathy (DN) by targeting fibrotic genes. The miR-let-7s family has been reported to have antifibrotic effects in DKD. Additionally, miR-24–3p regulates angiogenesis, wound healing, and fibrosis in DN, while miR-23a-3p inhibits the inflammatory response and fibrosis by targeting early growth response factor 1 (EGR1), which is involved in tissue injury in DN. Rats treated with urinary exosomes showed attenuated renal pathology, recovered expression of protective miRNAs, and improved renal function [63]. Furthermore, Jiang et al. illustrated that human urinary stem cell-derived exosomes (USCs-Exo), when injected into an in vivo model, could potentially reduce urinary microalbumin excretion, prevent podocyte and tubular epithelial cell apoptosis, suppress caspase-3 overexpression, and increase glomerular endothelial cell proliferation. In addition, USCs-Exo could reduce podocyte apoptosis induced by high glucose in vitro. These exosomes contained key factors, including growth factors, transforming growth factor-β1, angiogenin, and bone morphogenetic protein-7 (BMP-7), which may contribute to vascular regeneration and cell survival [64]. Moreover, urinary stem cell-derived exosomes (USCs-Exo) overexpressing miR-16–5p exhibited a renal protective effect by compensating the inhibited miR-16-5p due to high glucose and silencing vascular endothelial growth factor A (VEGFA), thereby improving podocyte injury [65].

Managing the cargo of exosomes that intracellular migrate in renal cells offers a promising therapeutic approach for DN through their ability to modulate key pathological pathways. Additionally, exosomes may act as a vehicle for drug delivery. The therapeutic effects of exosomes have become a focal point of interest; however, further studies are needed to confirm these findings and to optimize exosome-based therapies for clinical applications.

### Urinary exosomes as biomarkers in DN

There are several biomarkers for DN diagnosis; however, all commonly used biomarkers, such as ACR and eGFR, lack sensitivity and specificity. Until now, renal biopsy remains the definitive method for diagnosis of DN; however, its use is limited due to its invasive nature. Hence, identification of novel biomarkers for early diagnosis DN is crucial [66]. Urinary exosomes have emerged as promising biomarkers for early detection of DN. They can be easily obtained without invasive biopsy, produced from all kidney cells, which provide a complete overview about the urinary system [67, 68]. In hyperglycemic conditions, various signaling cascade pathways are activated that cause different gene and protein expression in the vesicles; therefore, the exosomes could reflect cells’ pathological and physiological state [69–71]. Furthermore, hypoxia-inducible factor-1, triggered in response to hypoxia during the initial stage of DN, stimulates exosome production from kidney tubules [36, 72]. Low-abundance urinary proteins are enriched in exosomes without contaminants. The cargo of exosomes (proteins, miRNA, mRNA) is more stable than other free-flowing molecules, as it is protected from degradation by the lipid bilayer of exosomes. Therefore, exosomes could provide diagnostic biomarkers without the need for invasive biopsy [73, 74]. However, the current methods for exosomes isolation and characterization often involve highly equipped, lengthy protocols, and costly instruments, such as ultracentrifuges, Immunoaffinity capture, and nanoparticle tracking analyzers [75, 76], and require highly skilled personnel to ensure reliable results. These challenges may limit the widespread use of exosomes as biomarkers. To address these challenges future studies are required to simplify the workflow and reduce costs of these methods to overcome the critical clinical translational for existing exosomal biomarkers and providing a framework for future discovery studies.

#### Proteins of urinary exosomes as biomarkers

Urinary exosomes are enriched with proteins, increasing the chance of detecting low-abundance proteins in urine. Consequently, several proteomic studies have been conducted to investigate the urinary exosomal protein as a protentional biomarker for DN (Table 2).

**Table 2:** Role of urinary exosomal protein as biomarkers for DN

Protein		Level	Type of diabetes	Species	No. of participants case/(control)	Detection method of exosomal protein	Comparing groups	Disease condition	Ref
TF	Transferrin	↑	Type 2	Human	15 DM 3, 36 DM2, 74 DM 1, 19 HC	LC-MS/MS Western blot	DKD (DM 3, DM 2) vs. DM 1 vs. HC	ACR> 30 mg/g	[77]
SERPINA1	Alpha-1 antitrypsin	↑							
AFM	Afamin	↑							
CTSD	Cathepsin D	↓							
PHYHD1	Phytanoyl-CoA dioxygenase domain containing 1	↑	Type 2	Human	20 DN, 20 NDRD, 16 T2DM, 21 HC	DIA-MS data acquisition. ELISA	DN vs. NDRD vs. T2DM vs. HC	Kidney biopsy	[78]
PAK6	Serine/threonine-protein kinase Epidermal growth factor receptor	↑ ↑	Type 2	Human	48/(48 T2DM, 48 HC)	LC-MS/MS Western blot ELISA	DN vs. T2DM and HC	ACR> 30 mg/g; eGFR < 60 ml/min/1.73m^2^	[79]
EGF	Epidermal growth factor receptor	↑							
AMBP MLL3	α-microglobulin/bikunin precursor Histone-lysine *N*-methyltransferase	↑ ↑	—–	Human	8/(8)	LC–MS/MS SRM	DN vs. HC	ACR> 30 mg/g. eGFR < 60 ml/min/1.73m2	[70]
MLL3	Histone-lysine *N*-methyltransferase	↑							
VDAC1	Voltage-dependent anion-selective channel protein 1	↓							
Regucalcin (SMP30)	Senescence marker protein-30	↓	Type 2	Rat Human	………. Urine 4/(3) Tissue 7/(4)	2-dimensional DIGE Western blot, IHC, SRM	STZ-induced diabetic rat with renal fibrosis vs. HC DN vs. HC	eGFR < 60 ml/min/1.73m^2^ Urine protein excretion of ≥ 150 mg/24 h	[80]
WT-1	Wilm’s tumor-1	↑	Type 1	Human	18/(30 DM, 25 HC) WT1 detected in 19 out of 31	Western blot Immunoblot	T1DM with proteinuria vs. T1DM without proteinuria and HC T1DM	ACR> 30 mg/g. Microalbumin >20 µg/min	[81] [82]
PEPD	Xaa-Pro dipeptidase	↑	Type 2	Rat	7/(7)	nLC-ESI-MS/MS Immunoblotting	ZDF rats sacrificed after 12 weeks, 20 weeks vs. lean control	......	[83]
MUP-1	Major Urinary Protein −1	↓							
AQP5, AQP2	Aquaporins	↑	Type 2	Human	12 DN, 12 DM, 11 NDN, 7 HC	ELISA Western blot	DN vs. DM vs. NDN vs. HC	Biopsy-proven	[84]
UMOD	Uromodulin	↑	Type 2	Human	22 Ma, 32 Mi, 46 No, 31 HC	ELISA	Ma vs. Mi vs. No vs. HC	ACR> 30 mg/g	[85]
CD 63		↓	—–	Human	8 Mi, 13 No	Flow cytometry	Mi vs. No	UAER from 30,300 mg/24 h	[86]
C-megalin		↑	Type 2	Human	19 Ma, 17 Mi, 20 No, 19 HC	Immunoblotting	Ma vs. Mi vs. No vs. HC	ACR> 30 mg/g	[87]

*HC* Healthy control, *DN* diabetic nephropathy, *DM* diabetes mellitus, *DKD* diabetic kidney disease, *ACR* albumin to creatinine ratio, *eGFR* estimated glomerular filtration rate, *AER* albumin excretion rate, *No* normoalbuminuria, *Mi* microalbuminuria, *Ma* macroalbuminuria, *SRM* selected reaction monitoring, *LC-MS/MS* liquid chromatography-tandem mass spectrometry, *DIGE* two-dimensional difference gel electrophoresis, *IHC* immunohistochemistry, *ZDF* Zucker diabetic fatty rats, *NDN* non-diabetic nephropathy

A recent proteomic study by Du et al. showed that levels of transferrin (TF), alpha-1 antitrypsin (SERPINA1), and afamin (AFM) increased with the progression of DKD, whereas levels of cathepsin D (CTSD) declined with DKD progression [77]. Moreover, another recent proteomic study by Ding and colleagues found that phytanoyl-CoA dioxygenase domain containing 1 (PHYHD1) was significantly increased in DN patients compared to non-diabetic renal disease (NDRD) and healthy controls (HC). This suggested PHYHD1 as a biomarker with high specificity that can contribute to differential diagnosis between DN and NDRD and reflect renal function and hyperglycemic management [78].

Li et al. recently demonstrated that serine/threonine-protein kinase (PAK6) and epidermal growth factor receptor (EGFR) were elevated significantly in DN compared to non-DN patients and healthy controls, indicating their potential as promising biomarkers for DN diagnosis [79]. Furthermore, a proteomic study by Zubiri et al. illustrated that α-microglobulin/bikunin precursor (AMBP) and histone-lysine N-methyltransferase (MLL3) were increased in DN, while voltage-dependent anion-selective channel protein 1 (VDAC1) were reported to be decreased [70]. Another study using a rat model, further confirmed by human urinary exosomal samples, showed that regucalcin protein, also known as senescence marker protein-30 (SMP30), which is involved in cellular Ca2+ homeostasis, the biosynthesis of ascorbate, and oxidative stress regulation, was downregulated in DN compared to healthy controls [80].

Wilm’s tumor-1 (WT-1), produced by podocytes and reflecting podocyte injury, was significantly increased in type 1 diabetic patients with proteinuria compared to those without proteinuria. WT-1 was associated with a renal function decline, suggesting its potential to predict early the risk of developing proteinuria in type 1 diabetic patients [81, 82]. Research conducted by Raimondo et al. on a type 2 diabetic rat model found that Xaa-Pro dipeptidase (PEPD), which is expressed in kidney tubules and plays an important role in the recycling of proline for collagen synthesis, was significantly increased in DN compared to the control and correlated with DN severity. Conversely, Major Urinary Protein-1 (MUP-1) was reduced in DN compared to control [83].

Aquaporins (AQPs), water membrane channels expressed in tubular epithelial cells, showed that the excretion of AQP5 and AQP2 positively correlated with the class of DN [84]. Uromodulin, a specific tubular protein produced only in the thick ascending limb of Henle’s loop, showed a progressive increase in DN 1 and DN 2 compared to the control group; thus, it may be used to predict the progression of DN [85]. The level of CD 63 was higher in normoalbuminuria than in microalbuminuria and declined significantly after α- lipoic acid (α‑LA) administration in normoalbuminuria patients. Therefore, a urinary CD63-positive exosome could be a potential sensitive and therapeutic indicator [86]. De et al. illustrated that C-megalin level increased with the progression of the albuminuric stages in patients with T2DM [87]. This highlight improving the detection of low-abundance proteins that may otherwise be difficult to identify in urine.

#### miRNA, mRNA of urinary exosomes as biomarkers

Urinary exosomes, which carry genetic information such as miRNA and mRNA, offer enhanced stability compared to free ones due to their protection from ribonucleases (RNases) by the lipid bilayer. Additionally, they are highly specific to kidney tissue, as they are not contaminated by miRNA that passes through the glomerular filtration barrier. Several studies have investigated the role of urinary exosomal transcriptomes in DN as summarized in Table 3.

**Table 3:** Role of urinary exosomal miRNA and mRNA as biomarkers for DN

		Level	Type of diabetes	Species	No. of participants case/(control)	Detection method of miRNA/mRNA	Comparing groups	Disease condition	Ref
**miRNA**	miR-145-5p, miR-27a-3p	↑	Type 2	Human	20/(20 DM, 20 HC)	RT-qPCR	DKD group vs. DM group and healthy control	UACR > 30 mg/g	[88]
	miRNA-615-3p	↑	Type 2	Human	42/(21 DM, 20 HC)	RT-qPCR.	DKD group vs. DM group and healthy control	UACR > 30 mg/g	[89]
	miR-92a-1-5p	↑	Type 2	Human	44/(36)	RNA sequencing, RT-qPCR	DN vs. healthy control	eGFR ≥ 30 ml/min/1.73m2	[38]
	miR663a	↓	Type 2	Human	5 PDKD, 4 NPDKD, 5 DM, 3 HC	RT-qPCR	PDKD vs. NPDKD vs. DM vs. HC	eGFR < 60 ml/min/1.73m2; 24-h urine protein excretion of ≥ 500 mg	[90]
	miR-103a-3p, miR-151a-5p, miR-191-5p, miR-1972, miR-22-3p, miR-24-3p, miR-26a-5p, miR-30d5p, miR-361-5p, miR-378a-3p, miR-4454, miR-200c-3p, miR-619-5p, let-7i-5p, miR-574-3p	↑	Type 2	Human	9/(9)	Microarray Analysis, Taqman qPCR	DN vs. DM without kidney disease	………	[63]
	miR-126, miR-155, miR-146	↑	Type 2	Human	30/(34 DM, 28 HC)	Taqman qPCR	Patients with albuminuria vs. healthy controls and patients without albuminuria.	Urinary albumin > 30 mg/L	[91]
	miR-4534	↑	Type 2	Human	17/(17)	Microarray Analysis, RT-qPCR	DKD vs. DM	Urinary microalbuminuria ≥300 mg/24 h	[92]
	miR-19b-3p	↑	Type 2	Human	28/(15)	RT-qPCR	DN vs. DM	Biopsy-proven ACR> 30 mg/g; eGFR < 60 ml/min/1.73m^2^	[39]
	miR-188-5p, miR-150-3p, miR-760, miR-3677-3p, miR-548ah-3p, miR-548p, miR-320e, and miR-23c	↑	—–	Human	6 DKD	Next-generation sequencing. (small RNA sequencing)	Nephrotic DKD at different stage, and non-diabetic CKD patients as control	UPCR > 300 mg/g	[93]
	miR-133a-3p and miR-153-3p	↓							
	miR-21-5p, let-7e-5p and miR-23b-3p	↑	Type 2	Human	14/(15)	RT-qPCR	DKD vs. DM with normal renal function	UACR > 3 mg/mmol; eGFR < 60 ml/min/1.73m^2^	[94]
	miR-30b-5p and miR-125b-5p	↓							
	let-7c-5p	↑	Type 2	Human	28/(20 DM; 15 HC)	RT-qPCR	DKD group vs. DM group and healthy control	ACR>25 mg/mmol; eGFR < 60 ml/min/1.73m^2^	[95]
	miR29c-5p and miR-15b-5p	↓							
	miR-362-3p, miR-877-3p, and miR-150-5p	↑	Type 2	Human	5/(5) Verification 20/(20)	RT-qPCR	DM with macroalbuminuria vs. DM with normoalbuminuria	ACR>25mg/mmol AER=300–800 mg/24 h eGFR < 60 ml/min/1.73m^2^	[96]
	miR-15a-5p	↓							
	miR-451-5p	↑	Type 1	Rat	…….	RT-qPCR	Diabetic rats after 6 weeks vs. 9 weeks vs. non-diabetic rats	…….	[97]
	miR-133b, miR-342, miR-30a	↑	Type 2	Human	44 Ma/66 Mi/56 No/54 HC	RT-qPCR	Ma vs. Mi vs. No vs. HC	UACR > 30 mg/g	[98]
	miR-15b, miR-34a, miR-636	↑	Type 2	Human	90/(46 DM; 44 HC)	Syber green-based PCR array; RT-qPCR	Patients with albuminuria vs. healthy controls and patients without albuminuria.	UACR > 30 μg/mg	[99]
	miR-320c	↑	Type 2	Human	(5 Mi; 3 No)/(8 DM; 8 HC) Verification 5 Mi/(6 DM; 6 HC)	RT-qPCR	DN group (No, Mi) vs. DM group and healthy control	UACR > 30 mg/g; eGFR < 60 ml/min/1.73m^2^	[49]
	miR-130a and miR-145	↑	Type 1	Human Mice	12/(12)	Taqman qPCR	Microalbuminuria vs. Normoalbuminuria Diabetic vs. non-diabetic mice	AER> 20 μg/min; ACR>25 mg/mmol	[100]
	miR-155 and miR-424 miR-145	↓ ↑							
	miR-144–3p, miR-26a-5p, and miR-30c-5p miR-31–5p, miR-200c-3p, and miR-671–5p	↑ ↑	Type 1	Human	8/(5) 17/(18)	Next-generation sequencing. (small RNA sequencing)	Overt vs. No PMA vs. IMA	AER> 200 μg/min AER> 20–200 μg/min;	[101]
	hsa-miR-320b, hsa-miR-30d-5p, hsa-miR-30e-3p, hsa-miR-30c-5p, hsa-miR-190a-5p, hsa-miR-29c-5p, hsa-miR-98–3p, hsa-miR-331–3p, hsa-let-7a-3p, hsa-miR-106b-3p, hsa-miR-30b-5p, hsa-miR-99b-5p, and hsa-let-7f-1–3p	↓	Type 1	Human	5/(4)	Paired-end sequencing (small RNA sequencing)	DKD group vs. healthy control	eGFR < 60 ml/min/1.73m^2^; UACR > 30 mg/g	[102]
**mRNA**	UMOD mRNA	↑	Type 2	Human	44 Ma/66 Mi/56 No/54 HC	RT-qPCR	Ma vs. Mi vs. No vs. HC	UACR > 30 mg/g	[68]
					19 Mild DKD; 15 sever DKD; 11 CKD; 166 DM;18 OC; 18 HC		Mild DKD vs. sever DKD vs. CKD vs. DM vs. OC vs. HC	ACR> 30 mg/g; eGFR < 60 ml/min/1.73m2	[103]
	SLC12A1 mRNA, NDUFB2 mRNA	↑	Type 2	Human	19 Mild DKD; 15 sever DKD; 11 CKD; 166 DM;18 OC; 18 HC	RT-qPCR	Mild DKD vs. sever DKD vs. CKD vs. DM vs. OC vs. HC	ACR> 30 mg/g; eGFR < 60 ml/min/1.73m2	[103]
	CCL21 mRNA	↑	Type 2	Human	32/(19 DM, 20 HC)	RT-qPCR	DN group vs. DM group and healthy control	Biopsy-proven UACR > 30 mg/g	[104]
	WT-1 mRNA	↑	Type 2	Human	10/(5)	RT-qPCR	Overt DN vs. healthy control	24h urinary protein> 3g/day	[105]
	MAP7, MSRB1, GPX3, IL32, NOX4, HRSP12, TINAG, CAPN3, CXCL14, MSRA, CRYAB, RBP5, and TMEM9	↑	Type 1	Human	17/(37)	Genome-wide sequencing (mRNA sequencing)	Macroalbuminuria vs. Normoalbuminuria	AER> 30 mg/day	[106]

*HC* Healthy control, *DN* diabetic nephropathy, *DM* diabetes mellitus, *PDKD* proteinuria diabetic kidney disease, *NPDKD* non-proteinuria diabetic kidney disease, *UACR* urinary albumin to creatinine ratio, *eGFR* estimated glomerular filtration rate, *AER* albumin excretion rate, *UPCR* urinary protein to creatinine ratio, *No* normoalbuminuria, *Mi* microalbuminuria, *Ma* macroalbuminuria, *PMA* persistent microalbuminuria, *IMA* intermittent microalbuminuria, *OC* obese control

Recent miRNA profiling showed that the levels of exosomal miR-145-5p and miR-27a-3p were significantly increased in the DKD group compared with the DM group and the control group, suggesting their potential as novel non-invasive diagnostic biomarkers for DKD [88]. Wang et al. recently illustrated that the expression level of urinary exosomal miRNA-615-3p was significantly higher in DKD patients than that in the control and the T2DM groups. It may be used as a novel biomarker for evaluating DKD progression [89]. Moreover, Tsai et al. identified a significant increase in urinary exosomal miR-92a-1-5p in DN, which is related to the pathogenesis of DN, suggesting it could predict kidney injury in type 2 diabetic patients [38].

A study by Sinha et al. illustrated that miR‑663a was downregulated in proteinuria DKD compared to non-proteinuria [90]. A recent panel by Mishra et al. revealed 15 miRNAs (miR-103a-3p, miR-151a-5p, miR-191-5p, miR-1972, miR-22-3p, miR-24-3p, miR-26a-5p, miR-30d5p, miR-361-5p, miR-378a-3p, miR-4454, miR-200c-3p, miR-619-5p, let-7i-5p, and miR-574-3p) were upregulated in type 2 DN compared to T2DM patients without kidney disease [63]. Gonzalez and colleagues found that miR-126 was significantly increased in diabetic patients with albuminuria compared to non-albuminuria and control groups, suggesting its potential for monitoring DKD progression. While the levels of miR-155 and miR-146 were increased in diabetic patients, with and without albuminuria, compared to control, indicating their potential for identifying individuals at risk for DKD [91].

The level of miR-4534 was up-regulated in DN and correlated with DKD. miR-4534 is involved in DKD progression and aggravating podocyte injury, thus suggesting it as a novel biomarker for type 2 DKD progression [92]. A study carried out on type 2 diabetic patients revealed a marked elevation of miR-19b-3p expression in urinary exosomes from DN patients compared to those with T2DM. It was positively correlated with the severity of albuminuria and associated with a marked tubulointerstitial inflammation; therefore, it may be used to predict the progression of DN [39]. Lee and colleagues identified significant upregulation of miR-188-5p, miR-150-3p, miR-760, miR-3677-3p, miR-548ah-3p, miR-548p, miR-320e, and miR-23c in DN patients. Conversely, miR-133a-3p and miR-153-3p showed significant downregulation in DN patients [93]. The expression levels of urinary exosomal miR-21-5p, let-7e-5p, and miR-23b-3p were significantly upregulated in type 2 DKD compared to type 2 with normal renal function, while miR-30b-5p and miR-125b-5p expression were significantly lower in type 2 DKD, these results confirmed by Zang et al. [94].

A study by Li and colleagues in type 2 diabetic patients reported that levels of let-7c-5p were significantly increased, whereas levels of miR29c-5p and miR-15b-5p were decreased, which are correlated with progression of DN and could predict DN [95]. Xie et al. revealed that miR-362-3p, miR-877-3p, and miR-150-5p were upregulated, while miR-15a-5p was downregulated in macroalbuminuria compared to normoalbuminuria [96]. Research using a type 1 diabetes rat model showed a substantial increase in miR-451-5p, suggesting it could be a sensitive predictor of DKD [97]. Eissa et al. showed significant increases in expression levels of miR-133b, miR-342, and miR-30a in type 2 DN compared to control [98]. Another study by Eissa and colleagues showed that miR-15b, miR-34a, and miR-636 were upregulated in type 2 DKD, indicating their potential as a novel diagnostic panel [99]. Expression level of miR-320c showed strong upregulation in macroalbuminuria and was associated with DN progression [49]. Research on type 1 diabetic patients and a mouse model showed that in patients the levels of miR-130a and miR-145 were significantly higher, while miR-155 and miR-424 levels were significantly lower in micro- than in normoalbuminuric patients; therefore, it may represent a novel candidate biomarker for DN [100]. Additionally, another genomic study on type 1 diabetic patients showed elevated level of changes of vesicles miR-144-3p, miR-26a-5p, and miR-30c-5p in macroalbuminuria compared to normoalbuminuric subject; moreover, it showed elevation of miR-31-5p, miR-200c-3p, and miR-671-5p in patients with persistent microalbuminuria compared to intermittent microalbuminuria patients [101]. A study on type 2 diabetic patients by Park et al. showed significant downregulation of 13 miRNAs were downregulated in DKD patients compared to control subjects (hsa-miR-320b, hsa-miR-30d-5p, hsa-miR-30e-3p, hsa-miR-30c-5p, hsa-miR-190a-5p, hsa-miR-29c-5p, hsa-miR-98-3p, hsa-miR-331-3p, hsa-let-7a-3p, hsa-miR-106b-3p, hsa-miR-30b-5p, hsa-miR-99b-5p, and hsa-let-7f-1-3p) [102].

mRNA of urinary exosomes has emerged as a potential biomarker for the progression of DN. Studies by Barr et al. and Yamamoto et al. revealed that the expression level of UMOD mRNA was significantly upregulated in patients with microalbuminuria compared to the healthy control group, indicating its potential to predict the early risk of developing proteinuria in type 2 diabetic patients [68,103]. Furthermore, Yamamoto and colleagues found that SLC12A1 mRNA and NDUFB2 mRNA levels were markedly increased in DKD patients compared to controls, and these levels were correlated with albuminuria, suggesting their utility as biomarkers for identifying DKD progression [103].

CCL21 mRNA, which is associated with the pathogenesis of DN by mediating T-cell infiltration and causing chronic inflammation, was significantly elevated in DN patients compared to healthy controls and T2DM patients, and its levels correlated with proteinuria. CCL21 mRNA serves as an early biomarker for identifying DN [104]. Additionally, a study by Abe et al. demonstrated that WT-1 mRNA level was upregulated in DN patients compared to healthy controls, reflecting progressive podocyte damage and predicting a decline in renal function [105]. A study by Dwivedi et al. on type1 DKD patients identified 13 mRNA genes (MAP7, MSRB1, GPX3, IL32, NOX4, HRSP12, TINAG, CAPN3, CXCL14, MSRA, CRYAB, RBP5, and TMEM9) that were significantly upregulated in macroalbuminuria compared to normoalbuminuria. Among them, six specific genes (GPX3, NOX4, MSRB, MSRA, HRSP12, and CRYAB) could identify individuals with normoalbuminuria who will experience early kidney function decline before traditional marker of DKD [106].

Urinary exosomes represent a non-invasive, stable, and highly informative source of biomarkers for DN, as their diverse cargo reflects the pathological state of their cells of origin. However, challenges in isolation and standardization need to be addressed to facilitate their widespread clinical use.

## Conclusion and perspective

Exosomes have shown great advancement as potential sources of pathophysiological information, future non-invasive diagnostic biomarkers, and therapeutic targets for DN as exosomes serve as mediators of intercellular communication among kidney cells via autocrine and paracrine signaling mechanisms. This is attributed to their stability, diverse cargo, and capability for intracellular crosstalk. Despite these advantages, several challenges remain, including the lack of established therapeutic strategies, standardized clinical guidelines, and a unified gold-standard method for exosome isolation. Although several isolation protocols such as ultracentrifugation, size-exclusion chromatography, and microfluidic-based separation have been proposed, they yield exosomes with varying purity and bioactivity. This underscores the need for a unified approach to exosome isolation, quantification, and functional assays to ensure reproducibility, comparability, and enhanced clinical applicability.

Further studies, including large-scale clinical trials, are required to validate the role of exosomes in the diagnosis and treatment of DN. Currently, WT-1 remains the only confirmed biomarker for early albuminuria prediction, while CD63 has demonstrated sensitivity as a therapeutic indicator.

Exosome-based therapeutic strategies, such as engineered exosomes loaded with therapeutic miRNAs, siRNAs, or anti-inflammatory agents, hold promise for targeting specific pathways involved in DN pathogenesis. However, additional research is needed to evaluate their safety, efficacy, biocompatibility, and feasibility for large-scale production.

Insights from clinical trials evaluating exosomes as biomarkers or drug delivery systems in cancer may provide valuable guidance for DN research. These studies have addressed critical translational challenges, including exosome stability, biodistribution, and therapeutic cargo optimization, which could be adapted to advance exosome-based applications in DN. Notably, cancer-derived exosomes influence disease progression by transferring bioactive molecules and facilitating immune evasion. Understanding these mechanisms in the context of DN could provide critical insights into how exosomes contribute to renal inflammation and fibrosis, potentially guiding the development of targeted exosome-based therapeutic strategies.

## Data Availability

No datasets were generated or analysed during the current stud.

## Declarations

### Submission declaration

The authors declared that this manuscript hadn’t been published previously. If it is accepted, it will not be published elsewhere.

## Abbreviation

ARF6: ADP-ribosylation F6
ADMSCs: Adipose mesenchymal stem cell
ADSCs: Adipose stem cells
AFM: Afamin
ALIX: ALG-2 interacting protein
AMBP: α-microglobulin/bikunin precursor
ATF-6: Activating transcription factor 6
BMMSC: Bone marrow mesenchymal stem cells
CTSD: Cathepsin D
DUSP1: Dual specificity phosphatase 1
EGR1: Early growth response factor 1
EMT: Epithelial-mesenchymal transition
ERBB3: Erb-b2 receptor tyrosine kinase 3
ERK: Extracellular signal-regulated kinase
ESCRT: Endosomal sorting complex required for transport
ET-1: Endothelin-1
GECs: Glomerular endothelial cells
GMCs: Glomerular mesangial cells
HDAC1: Histone deacetylase-1
HG: High glucose
HUC-MSCs: Human umbilical cord mesenchymal stem cells
ILVs: Inter luminal vesicles
IPA: Ingenuity Pathway Analysis
LAMP-3: Lysosomal-associated membrane protein 3
LMP1: Latent membrane protein 1
MCs: Mesangial cells
MLK: Myosin light chain kinase
MMP-2: Matrix metalloproteinase-2
MPCs: Mesangial podocyte cells
MSCs: Mesenchymal stem cells
MUP-1: Major Urinary Protein -1
MVBs: Multi vesicular bodies
NG: Normal glucose
nSMase: Neutral sphingomyelinase
PAK6: Serine/threonine-protein kinase
PEPD: Xaa-Pro dipeptidase
PHYHD1: Phytanoyl-CoA dioxygenase domain containing 1
PLD: Phospholipase-D
PMEL: Melanocyte-specific glycoprotein
PTECs: Proximal tubular epithelia cells
RAS: Renin-angiotensin system
RCN3: Reticulocalbin3
RNases: Ribonucleases
ROS: Reactive oxygen species
Srgap2: Slit-Roundabout (Slit-Robo) GTPase-activating protein 2
STAM-1: Signal transduction adaptor molecule-1 protein
STATA1: Signal transducer and activator of transcription
TEC: Tubular epithelial cell
TEMs: Tetraspanin-enriched microdomains
TGβ-1: With transforming growth factor beta
TRAF6: Tumor necrosis factor receptor-associated factor-6
TSG: Tumor susceptibility gene
TSP-1: Thrombospondin-1
VDAC1: Voltage-dependent anion-selective channel protein 1
VEGFA: Vascular endothelial growth factor A
WT-1: Wilm’s tumor-1
YAP1: Yes- associated protein 1
ZEB2: Zinc finger E-box-binding homebox-2
α-LA: α- lipoic acid
